# The Comprehension, Cosmetics, Convenience, Content, and Credibility of Infographic Patient Information Leaflets (iPILs) Compared to Existing PILs (ePILs)

**DOI:** 10.3390/healthcare13111227

**Published:** 2025-05-23

**Authors:** Xin Pan, Eunhee Kim, Jose Zamora, Micah Hata, Andrea Wooley, Radhika Devraj, Hyma P. Gogineni, Anandi V. Law

**Affiliations:** 1 College of Pharmacy, Western University of Health Sciences, Pomona, CA 91766, USA; eunhee.kim@westernu.edu (E.K.); jzamora@westernu.edu (J.Z.); mhata@westernu.edu (M.H.); hgogineni@westernu.edu (H.P.G.); alaw@westernu.edu (A.V.L.); 2 Department of Pharmaceutical Sciences, School of Pharmacy, Southern Illinois University Edwardsville, Edwardsville, IL 62025, USA; abasso@siue.edu (A.W.); rdevraj@siue.edu (R.D.)

**Keywords:** health literacy, patient information leaflets, patient medication information, patient preferences, patient education, medication safety

## Abstract

**Background/Objectives**: Existing patient information leaflets (ePILs), mandated by the FDA to accompany new prescriptions, are difficult to read and understand due to their complexity and poor visual design, especially for populations with low health literacy and low English proficiency. In this study, we developed infographic-based PILs (iPILs) with a concise question-and-answer format, emphasizing essential information, as specified by the FDA. This study compared iPILs and ePILs using the 5C factors: comprehension, cosmetics, convenience, content, and credibility, as perceived by English-speaking and Spanish-speaking populations. **Methods**: This multicenter, experimental survey study assessed the 5C factors. English and Spanish-speaking adults on ≥1 chronic medication were recruited from community pharmacies in California (CA) and Illinois (IL). They were stratified to review either an ePIL or an iPIL for one of four common medications. They completed a Medication Knowledge Quiz (MKQ) to show their comprehension using six open-ended questions. Subsequently, they received both PIL versions and answered preference questions about the 4C and media format and, lastly, about demographic and health literacy questions. **Results**: A total of 235 participants completed the surveys at three sites (CA-English, CA-Spanish, and IL-English), with differing participant characteristics. The CA-Spanish participants scored the lowest on health literacy and the number of health conditions. The MKQ scores for those using the iPILs were significantly higher than for those using the ePILs across all groups. They significantly correlated with health literacy results for the ePILs (r = 0.394, *p* < 0.001). The participants preferred the iPILs over the ePILs for four of the C factors, barring one content question. Regardless of age, printed formats were preferred (64.7%)—alone or with digital formats (21.3%)—over digital formats alone (3.4%). Overall, 79.1% of the participants preferred iPILs, 11.9% preferred ePILs, and 8.9% preferred either version. **Conclusions**: The infographic-based patient information leaflets (iPILs) were easier to read, navigate, and understand, making them more accessible to individuals with varying levels of health literacy. Infographic-based leaflets outperformed existing ones in user comprehension and were preferred due to their simple layout, ease of navigation, and helpfulness.

## 1. Introduction

Patient information leaflets (PILs), also known as patient medication information (PMI) leaflets, are informative paper handouts. These are mandated by the Food and Drug Administration (FDA) and must be distributed alongside prescription medications [1]. Unlike the patient package inserts generated by drug manufacturers, PILs are created by pharmacies or private vendors and are not reviewed or approved by the FDA. However, the FDA has established specific content and format guidelines to ensure accuracy and clarity. PILs typically cover essential medication details, including proper usage, adverse effects, storage, precautions and warnings, and patient counseling information [2].

While PILs have been a credible source of medication information, their complexity and poor visual presentation can pose challenges for patients in reading and understanding, especially among vulnerable populations such as older populations and those with low English proficiency (LEP) and/or health literacy [1,3,4,5,6]. A previous study, where patients were given PILs associated with new or refill prescriptions, found that 96.6% of the respondents expressed concerns regarding the understandability of the leaflets and 96.3% questioned their usefulness [1]. Healthy People 2030 has identified health literacy as a priority area, defining it as the extent to which individuals can find, comprehend, and apply information and services to make informed health-related choices [7,8].

Research has also shown that PILs are rarely or never read by approximately 30% of patients receiving new medications and 65% of patients receiving refilled medications due to a lack of comprehension [9]. These challenges often stem from complex language, small font sizes, confusing layout, and busy formats [3,10]. Therefore, the usability of PILs was found to be low and could be correlated with the unintentional incorrect usage of medications, encompassing medication underuse, overuse, and misuse [3,4,5,6]. These, in turn, could lead to medication-related misadventures, contributing to both health and economic burdens [5,6,11,12].

The aforementioned factors pointed to a need for PILs that can demystify complex medical information by using a visually engaging and easily understandable format for a wide range of health literacy levels. The use of infographics could effectively capture patients’ attention by offering increased access to visual instructions, as well as highlighting the most relevant information to the patients.

Previous research from our team has included redesigning prescription labels using three parameters: simplified content, convenience of finding information, and cosmetic or appealing design [13,14]. Studies testing these redesigned labels against existing labels showed the redesigned label to be more user-friendly; easier to understand; and preferred, overall, by patients, pharmacists, and physicians, regardless of users’ education and skill levels.

In the current study, we used a similar approach with the 5C factors: comprehension, cosmetics, convenience, content, and credibility. Comprehension refers to how easily the material can be understood by the user. Cosmetics focus on the visual appeal, including elements such as font type, font size, and overall layout design. Convenience refers to the ease of use and smooth navigation to quickly locate relevant information. Content addresses the clarity, organization, and level of detail provided in the material. Credibility highlights the trustworthiness of the information presented. Infographic-based PILs (iPILs) were designed to address the 5C factors with a concise Q&A format and limited wording, focusing on the essential information specified by the FDA.

The goal of this study was to compare the perception of iPILs and ePILs across the 5C factors—comprehension, cosmetics, convenience, content, and credibility—in English-speaking and Spanish-speaking populations. Our hypothesis was that iPILs would score higher in comprehension and be preferred over ePILs in terms of cosmetics, convenience, content, and credibility.

## 2. Materials and Methods

### 2.1. Study Design

This study was a multicenter experimental study, which was conducted from April 2023 to June 2024. It used an in-person administered survey to assess the 5C factors (comprehension, cosmetics, convenience, content, and credibility).

The study protocol was approved by the Institutional Review Board at Western University of Health Sciences (ID: 2041762) and Southern Illinois University Edwardsville (ID: 2156). Eligible participants provided informed consent upon reviewing the survey’s introduction. This study adhered to the Strengthening the Reporting of Observational Studies in Epidemiology (STROBE) guidelines for reporting cross-sectional research [15].

### 2.2. Sampling

English-speaking adults who were taking at least one chronic medication were recruited from local independent pharmacies: 986 Pharmacy in La Verne, CA (CA-English) and Medicate Pharmacy in East St. Louis, IL (IL-English). Spanish-speaking participants were recruited from another 986 Pharmacy located in El Monte, CA (CA-Spanish).

Individuals under the age of 18 or taking only acute or short-term medication were excluded. Data collectors set up a table at these pharmacies and approached patrons who were waiting for prescriptions or vaccinations. These patrons were informed about the study and invited to participate.

The desired sample size was 64 participants in each group (ePILs vs. iPILs), determined by an independent *t*-test, with a statistical power of 0.8, an alpha level of 0.05, and a medium effect size (d = 0.5) using G*Power 3.1 [16].

### 2.3. Data Collection Tool and Infographic-Based PILs (iPILs)

Prior studies in label redesign, cosmetics, convenience, and content were used as a framework to develop iPILs for four commonly used prescription medications: (atorvastatin, lisinopril, metformin, and sertraline [15,16]. The content in these iPILs was simplified and presented in a concise Q&A format, at a fifth grade reading level, focusing on the essential information specified by the FDA.

The study utilized a thirty-one-item survey that was developed by the research team. This survey included a six-item open-ended Medication Knowledge Quiz (MKQ) to assess comprehension, eleven preference questions to evaluate preferences between the two PIL versions across four factors (cosmetics, convenience, content, and credibility), media format and overall preference, and fourteen questions related to participant characteristics such as health literacy and demographics. Preferences for each PIL versions were assessed using categorical response options (Version 1, Version 2, Both, and Neither).

The four health literacy questions were created by modifying an existing validated BRIEF Health Literacy tool, as the original tool was designed for hospital settings and not applicable to our community-based sample population [17]. Face and content validation were conducted with ten experts and ten laypeople to ensure the accuracy and clarity of the iPILs and survey. The feedback was generally positive, with only minor changes recommended to simplify wording. Four MKQ items were adjusted to improve clarity and we specified the answer choices for the health literacy questions.

### 2.4. Data Collection

**Figure 1:**
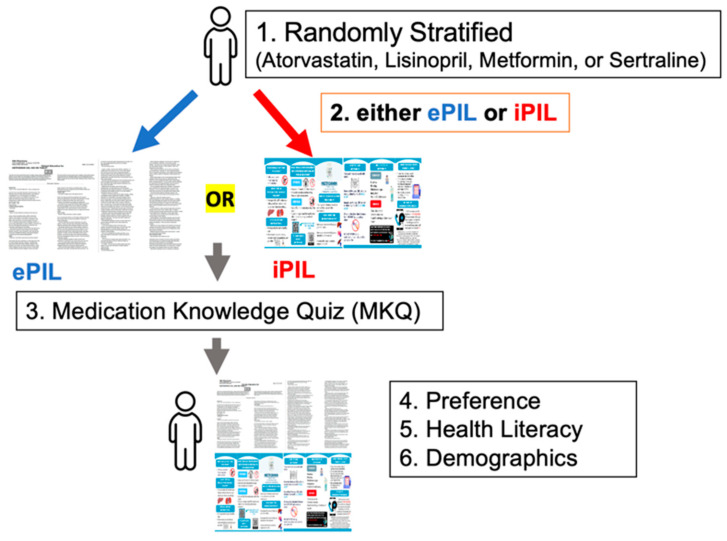
Data collection process.

As indicated in Figure 1, the participants were stratified to start with either the ePIL or iPIL version for one of the four medications (atorvastatin, lisinopril, metformin, or sertraline). Participants were assigned to either the ePIL or iPIL version using a systematic alternating sequence to ensure a balanced distribution across the two formats. Subsequently, one of the four medications was assigned using a predetermined sequence to support unbiased allocation across medication groups.

Following a review of the assigned PIL version, participants took a Medication Knowledge Quiz (MKQ), with six open-ended questions. They answered these questions based on the information found in the PIL version provided, resulting in an overall comprehension score.

Subsequently, they received the other PIL version and, with both versions to hand, completed questions evaluating the cosmetics, convenience, content, and credibility of each PIL version. Lastly, the participants responded to questions on preference and participant characteristics.

If a participant agreed to complete the survey for a second medication, the process was repeated, beginning with the other PIL version.

### 2.5. Outcome Measures

The comprehension score (0–6) was calculated as the sum of six items from the MKQ, with completely correct answers scored as 1, partially correct as 0.5, and incorrect as 0. A higher comprehension score reflected better content understanding. The data collectors (student pharmacists on rotation or pharmacy residents) underwent training on the clearly defined criteria for scoring the six MKQ items. A single researcher trained each data collector, ensuring consistency. Sample scoring was used to test the data collectors on correct and partially correct answers, helping minimize inter-rater differences.

Since these responses were open-ended and free text, ‘partially correct’ indicated that the responses were accurate to some extent, but not fully correct. For example, when asked about the storage of metformin, a ‘partially correct’ answer might be ‘keep out of reach of children’, while a ‘fully correct’ answer would include ‘keep out of reach of children and pets, and store at room temperature, away from moisture’.

The health literacy score (4–16) was derived from four questions on a 4-point Likert scale (1 = all of the time, 2 = most of the time, 3 = some of the time, and 4 = never). These questions were adapted by modifying the validated BRIEF Health Literacy tool [17]. The scoring guidelines provided by this tool were used for a cut-off score of nine to distinguish between low and high health literacy. Health literacy scores of 4–9 indicated low health literacy, while scores of 10–16 indicated high health literacy.

### 2.6. Statistical Analysis

Data were collected through Microsoft Excel or the Qualtrics platform. A descriptive analysis, including means with standard deviations and counts with percentages, was performed to summarize survey responses [18]. One-way ANOVA with Tukey’s post hoc tests (continuous variables) and chi-squared tests (categorical variables) were used to compare scores and characteristics among groups. A correlational analysis assessed the association between comprehension and health literacy scores. Linear regression models were performed to evaluate the impact of the PIL version and language on comprehension scores. To control for potential confounders, we adjusted for demographic characteristics: age, gender, race/ethnicity, education level, annual household income, and insurance type, health literacy, and prior experience with medication use. A reliability analysis utilizing Cronbach’s alpha was used to determine internal consistency. All analyses were conducted using IBM SPSS v29.0 at a 95% significance level [19].

## 3. Results

### 3.1. Participant Characteristics

A total of 235 participants completed the survey between March 2023 and May 2024 at three different sites: 986 Pharmacy in La Verne, CA (CA-English, n = 105); 986 Pharmacy in El Monte, CA (CA-Spanish, n = 68); and Medicate Pharmacy in East St. Louis, IL (IL-English, n = 62). Participants’ baseline characteristics are shown in Table 1. The key characteristics were different between the three groups, except for gender. The participants from the CA-English group were significantly older, white in ethnicity, and had higher education levels and annual incomes compared to the other two groups. The participants from the CA-Spanish group had significantly fewer health conditions, prescription medications, and lower health literacy than the other two groups.

### 3.2. Medication Knowledge Quiz Assessing Comprehension

Table 2 summarizes the accuracy rate for each item in the Medication Knowledge Quiz and the overall comprehension score (mean total score of the MKQ) for both the iPIL and ePIL format. It also presents a subgroup analysis across the three pharmacy groups. Generally, the accuracy rates for the iPIL format were higher than those for the ePIL format across almost all items. All three groups showed significantly higher accuracy rates for the question about what to avoid (item five). The CA-English group had a significantly higher accuracy rate on medication precautions (item three), while the IL-English and CA-Spanish groups had significantly higher accuracy rates on side effects (item two).

Overall, the comprehension scores for the iPILs were significantly higher than for the ePILs across all groups, with IL-English achieving the highest scores in both comprehension and health literacy. A pooled analysis for all three sites demonstrated that the iPILs had significantly higher comprehension scores than the ePILs (5.05 vs. 3.96, *p* < 0.001). The participants’ previous experience with medication use did not differ significantly between the two groups (iPILs vs. ePILs) (*p* = 0.123).

### 3.3. Association Between Comprehension Score and Health Literacy

The four items on health literacy had a Cronbach’s coefficient alpha of 0.80, indicating high internal consistency.

No significant differences in health literacy were noted between the iPILs and ePILs (12.72 vs. 12.78, *p* = 0.897). As shown in Table 2, the comprehension score and health literacy were significantly correlated for the ePILs (r = 0.394, *p* < 0.001) but not for the iPILs (r = 0.128, *p* = 0.161). The exception was the correlation between the comprehension score for the ePILs and the health literacy score for the CA-Spanish group (r = 0.177, *p* = 0.308). IL-English had the highest health literacy and comprehension scores, which was consistent with our finding of a positive correlation between the two.

### 3.4. Associations of Comprehension Score with PIL Version and Language

Participants who used an iPIL had a significantly higher comprehension scores (1.09 points higher) than those who used an ePIL. This positive association persisted even after adjusting for confounding factors (demographic characteristics, health literacy, and prior experience with medication use), as shown in Table 3. However, no association was noted between the comprehension score and the language, even after the adjustment.

### 3.5. Preference Across Four Factors: Cosmetics, Convenience, Content, and Credibility

**Figure 2:**
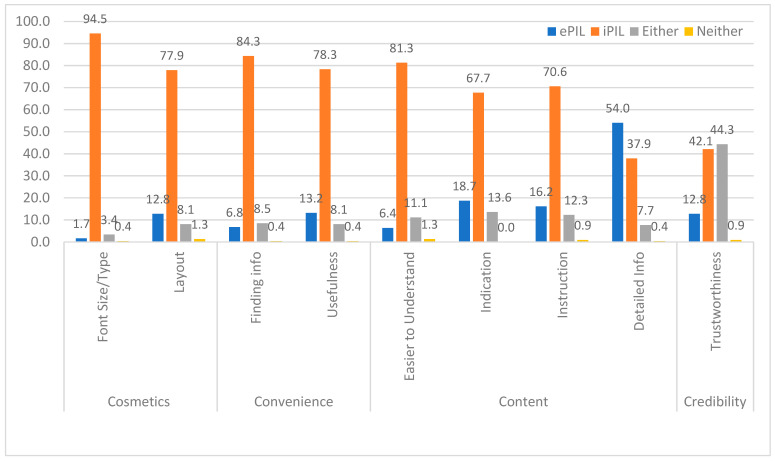
Comparison of PIL versions on preference domains (%).

A pooled analysis was conducted for the preference questions, assessing four factors: cosmetics, convenience, content, and credibility. As indicated in Figure 2, regardless of the order of presentation of the different PIL versions, the preference was much higher for the iPILs (42.1–94.5%) than for the ePILs (1.7–54.0%) across all four factors. This was except for the content question, which asked which version provided more detailed information. While 44% of the participants found both PIL versions equally trustworthy, a higher percentage of them preferred the iPIL to the ePIL alone (42.1% vs. 12.8%).

A subgroup analysis showed that the CA-English group followed the same pattern as the pooled analysis results, while the IL-English and CA-Spanish groups had a stronger preference for iPILs across all four factors.

The participants reported an overall preference for iPILs (79.1%) over ePILs (11.9%), with a smaller percentage (8.9%) preferring either version. Additionally, the preference was for either just the printed format (64.7%) or for both the printed and digital formats (21.3%) over the digital format alone (3.4%), regardless of age.

## 4. Discussion

This study examined and compared the 5C factors (comprehension, cosmetics, convenience, content, and credibility) of iPILs and ePILs using an in-person, administered survey tool. The study results revealed that the iPILs developed by our team outperformed the ePILs across all 5C factors.

The respondent characteristics were different among the three groups: the IL-English and CA-Spanish groups had a higher proportion of participants who were younger, with lower education levels, and lower annual incomes. However, the response patterns were broadly consistent across the 5C factors. Therefore, pooled analyses were conducted to better understand the overall trends and patient preferences.

Compared to information-dense ePILs, the iPILs appeared to enhance usability with simply worded content in a Q&A format, while maintaining credibility. Across all three groups and in the regression analysis, the iPILs were significantly easier to understand, as evidenced by the notably higher comprehension scores than those for the ePILs. Our findings closely align with previous studies, which demonstrated that simplifying PILs with straightforward language and an easy-to-navigate layout can improve patients’ comprehension of medication information [5,6,20].

The comprehension scores for the ePILs were significantly positively correlated with the participants’ health literacy scores, suggesting that only those with high health literacy levels could fully understand and digest the complex content of the ePILs. In contrast, the iPILs stood out for their simplicity and better accessibility, even after accounting for individuals’ education levels, health literacy, and prior experience with medication use. As a result, iPILs may be particularly beneficial in helping vulnerable populations better understand medication information [5,6].

Overall, the participants expressed a stronger preference for the iPILs due to several factors: (1) their superior visual appeal (cosmetics), including comfortable font size and an easy-to-follow layout; (2) ease of information retrieval, facilitated by a straightforward question-and-answer (Q&A) format and explanatory pictures (convenience); and (3) clear presentation of medication information, avoiding complicated medical terminology and considering the needs of patients with low health literacy or limited English proficiency (content). By making critical medication information more understandable and accessible, iPILs have the potential to positively impact patient outcomes and adherence to prescribed therapies [5,6,20,21,22].

While both the iPILs and ePILs had similar levels of credibility, most participants believed that the ePILs offered more detailed information, whereas iPILs were simpler to read, navigate, and understand. Providing overly detailed information is not necessarily helpful for patients as it is often confusing and the language presented may compound patient fears related to potential side effects and warnings [21,22]. These effects can be intensified in vulnerable populations, such as older populations and those with low English proficiency (LEP) and/or low health literacy [1,3,4,5,6]. It is important that information should be pertinent and concise, as excessive details can overwhelm patients and make it difficult for them to find relevant information.

Given the concerns about the considerable paper waste associated with paper format PILs, it is worth highlighting that the participants across all age groups in our study preferred receiving either printed PILs or a combination of printed and digital PILs over digital PILs alone. Despite the rampant use of digital formats, such as QR codes, our inquiry into participants’ preferences revealed that these were not favored by our predominantly elderly sample. Further investigation is needed in this area.

In 2023, the FDA proposed a rule to create a new type of patient medication information, one which is more concise, accessible, and simplified to help patients use their prescription medications safely and effectively [23]. Our iPILs were designed to be streamlined and easy to navigate, catering for diverse learning styles and health literacy levels, making medication information more accessible and inclusive. The implementation of iPILs, combined with efforts to enhance prescription medication labels, has the potential to significantly improve patients’ ability to obtain, understand, and process critical medication information. By enhancing patients’ comprehension of medication information, it can promote better self-care practices; reduce medication administration errors; and, ultimately, improve medication adherence [10,13]. These improvements are particularly crucial for individuals managing complex medication regimens or those with limited health literacy or low English proficiency, as they support safer and more effective treatment outcomes [5,6,22].

A limitation of our study was the potential for inconsistencies due to the data collection being undertaken across multiple sites and by different collectors. However, standardized data collection protocols were provided to minimize this risk. Another limitation, as is true of all survey studies, was the reliance on self-reported data for the health literacy questions, which may have introduced bias or inaccuracies in reporting health literacy. The lack of generalizability was another limitation of this study. Recruitment was confined to independent pharmacies in California and Illinois, which may not have reflected broader geographic or healthcare settings. Additionally, the sample lacked age diversity, as most participants were older adults. Furthermore, our iPILs focused on only four commonly used oral medications, limiting the applicability of our findings to medications administered via different routes, such as inhalers and injections. Another limitation was that the participants who completed the survey for a second medication using the other PIL version were counted twice in the sample size. This applied to only 14 participants (14/235 = 6%) from the CA-English group, but there may have been the potential for non-independence in responses to the preference questions.

Future studies could explore these findings in diverse settings, populations, and languages to broaden our understanding of the usability of iPILs and their long-term effects on medication adherence and clinical outcomes. Additionally, employing longitudinal or experimental designs along with cost-effectiveness analyses would provide insights into the feasibility and broader adoption of iPILs in clinical practice. Finally, exploring the potential for digital adaptation and integration into electronic health records systems for better patient education could be another valuable direction for future research.

## 5. Conclusions

This study examined the 5C factors (comprehension, cosmetics, convenience, content, and credibility) of infographic-based patient information leaflets (iPILs) compared to existing PILs (ePILs) using an in-person, administered survey tool. In contrast to the complex and visually challenging ePILs, our iPILs enhanced usability with simple content in a Q&A format; improved comprehension without compromising credibility; and were easier to read, navigate, and understand. Our study found that iPILs were preferred and more accessible to individuals with various health literacy levels.

## Figures and Tables

**Table 1:** Baseline characteristics of patient groups.

Characteristics	CA-English	IL-English	CA-Spanish	*p*-Value
	(n = 105)	(n = 62)	(n = 68)	
**Age**, n (%)				
18–34	1 (1.0%)	13 (21.0%)	9 (13.2%)	<0.001
35–54	31 (29.5%)	24 (38.7%)	33 (48.5%)	
54+	73 (69.5%)	22 (35.5%)	26 (38.2%)	
Prefer not to disclose	0 (0%)	3 (4.8%)	0 (0%)	
**Gender**, n (%)				
Male	35 (33.3%)	22 (35.5%)	21 (30.9%)	0.191
Female	70 (66.7%)	38 (61.3%)	47 (69.1%)	
Prefer not to disclose	0 (0%)	2 (3.2%)	0 (0%)	
**Ethnicity**, n (%)				
White	38 (36.2%)	9 (15.5%)	0 (0%)	<0.001
Black	13 (12.4%)	44 (75.9%)	0 (0%)	
Hispanic	38 (36.2%)	3 (5.2%)	68 (100%)	
Asian	8 (7.6%)	0 (0%)	0 (0%)	
American Indian/Alaska native	2 (1.9%)	0 (0%)	0 (0%)	
Hawaiian/Pacific Islander	1 (1.0%)	0 (0%)	0 (0%)	
Other	4 (3.8%)	1 (1.7%)	0 (0%)	
Prefer not to disclose	1 (1.0%)	1 (1.7%)	0 (0%)	
Missing	0	4	0	
**Education level**, n (%)				
Less than high school	2 (1.9%)	5 (8.1%)	22 (32.4%)	<0.001
High school diploma	17 (16.2%)	30 (48.4%)	31 (45.6%)	
Some college	49 (46.7%)	17 (27.4%)	10 (14.7%)	
Bachelor’s degree	22 (21.0%)	4 (6.5%)	2 (2.9%)	
Master’s degree or higher	14 (13.3%)	0 (0%)	2 (2.9%)	
Other/prefer not to disclose	1 (1.0%)	6 (9.7%)	1 (1.5%)	
**Annual income**, n (%)				
Disabled/unemployed	0 (0%)	0 (0%)	6 (8.8%)	<0.001
≤$25,000	19 (18.1%)	18 (29.0%)	18 (26.5%)	
$25,000–49,999	29 (27.6%)	17 (27.4%)	29 (4.6%)	
$50,000–99,999	20 (19.0%)	5 (8.1%)	4 (5.9%)	
$100,000–149,999	13 (12.4%)	1 (1.6%)	1 (1.5%)	
≥$150,000	13 (12.4%)	0 (0%)	0 (0%)	
Prefer not to disclose	11 (10.5%)	21 (33.9%)	10 (14.7%)	
**Insurance plan type**, n (%)				
None	1 (1.0%)	0 (0%)	8 (11.8%)	<0.001
Medicare only	7 (6.7%)	4 (6.5%)	15 (22.1%)	
Medicaid only	5 (4.8%)	20 (32.3%)	23 (33.8%)	
Both Medicare and Medicaid	24 (22.9%)	1 (1.6%)	8 (11.8%)	
Private insurance only	44 (41.9%)	24 (38.7%)	13 (19.1%)	
Both private and Medicare	16 (15.2%)	0 (0%)	0 (0%)	
Other	5 (4.8%)	1 (1.6%)	1 (1.5%)	
Not sure	3 (2.9%)	12 (19.4%)	0 (0%)	
**Number of health conditions** (mean ± SD)	2.08 ± 1.29	2.41 ± 1.07	1.47 ± 1.06	<0.001
**Number of prescription medications**, n (%)				
1–2 med(s)	35 (33.3%)	14 (23.0%)	43 (63.2%)	<0.001
3–5 meds	44 (41.9%)	33 (54.1%)	20 (29.4%)	
6–10 meds	19 (18.1%)	13 (21.3%)	5 (7.4%)	
More than 10 meds	7 (6.7%)	1 (1.6%)	0 (0%)	
Missing	0	1	0	
**Health literacy score** (mean ± SD)	13.35 ± 2.75	13.79 ± 2.27	10.87 ± 2.82	<0.001
**Health literacy**, n (%) Low health literacy (score 4–9)	8 (7.6%)	3 (4.8%)	23 (33.8%)	<0.001
High health literacy (score 10–16)	97 (92.4%)	59 (95.2%)	45 (66.2%)	

SD—standard deviation.

**Table 2:** Medication knowledge quiz scores and correlation with health literacy.

Quiz Item		Accuracy Rate ^a^ (%)					
		CA-English		IL-English		CA-Spanish	
		ePIL	iPIL	ePIL	iPIL	ePIL	iPIL
		(n = 49)	(n = 56)	(n = 29)	(n = 33)	(n = 35)	(n = 33)
1	One health condition this medication is used for	89.8	96.4	87.2	93.9	77.1	93.9
2	One side effect of this medication	75.5	80.4	72.4 *	100.0 *	60.0 *	84.8 *
3	One precaution while taking this medication	14.3 *	50.0 *	51.7	78.8	51.4	61.8
4	Side effect needing immediate medical attention	63.3	60.7	72.4	87.9	31.4 *	72.7 *
5	What to avoid while taking this medication	26.5 *	83.9 *	39.3 *	84.8 *	42.9 *	75.8 *
6	How this medication should be stored	87.8	87.5	86.2 *	100.0 *	82.9	87.9
**Comprehension score ^b^** (mean ± standard deviation)		3.97 ± 1.28 *	4.81 ± 1.10 *	4.36 ± 1.78 *	5.61 ± 0.67 *	3.57 ± 1.41 *	4.91 ± 1.52 *
**Pooled analysis**		**ePIL (n = 113)**			**iPIL (n = 122)**		
		3.95 ± 1.48 *			5.05 ± 1.18 *		
**Correlation with health literacy** (r)		0.338 *	−0.003	0.654 *	0.340	0.177	0.100
**Pooled analysis**		**ePIL (n = 113)**			**iPIL (n = 122)**		
		0.394 *			0.128		

^a^ Percentage of fully correct responses. ^b^ Included incorrect, partially, and fully correct scores. * *p* < 0.05.

**Table 3:** Association between PIL version, language, and mean comprehension score.

	Mean Comprehension Score			
	Mean Difference (95% CI)	*p*-Value	Adjusted Mean Diff. * (95% CI)	*p*-Value *
**PIL version**				
ePIL	Reference		Reference	
**iPIL**	1.09 (0.75–1.43)	<0.001	1.07 (0.74–1.40)	<0.001
**Language**				
English	Reference		Reference	
Spanish	−0.37 (−0.75–0.01)	0.053	−0.05 (−0.51–0.41)	0.840

Mean Diff.—mean difference; CI—confidence interval. * Adjustment for demographic characteristics (age, gender, race/ethnicity, education level, annual household income, and insurance type); health literacy; and prior experience with medication use.

## Data Availability

The original contributions presented in this study are included in the article. Further inquiries can be directed to the corresponding author.

## Abbreviations

The following abbreviations are used in this manuscript:

PILs	Patient Information Leaflets
iPILs	Infographic-based Patient Information Leaflets
ePILs	Existing Patient Information Leaflets
PMI	Patient Medication Information
FDA	Food and Drug Administration
LEP	Low English Proficiency
Q&A	Question and Answer
CA	California
IL	Illinois
MKQ	Medication Knowledge Quiz
ANOVA	Analysis of Variance
SD	Standard Deviation
